# A Smart Insole to Promote Healthy Aging for Frail Elderly Individuals: Specifications, Design, and Preliminary Results

**DOI:** 10.2196/rehab.4084

**Published:** 2015-05-25

**Authors:** Antoine Piau, Yoann Charlon, Eric Campo, Bruno Vellas, Fati Nourhashemi

**Affiliations:** ^1^CHU de ToulouseGerontopôleUPSF-31400 ToulouseFrance; ^2^CNRSLAASF-31400 ToulouseFrance; ^3^Univ de ToulouseUT2JLAASF-31100 ToulouseFrance; ^4^INSERMUMR 1027UPSF-31400 ToulouseFrance

**Keywords:** frail elderly, gait, healthy aging, wearable sensors

## Abstract

**Background:**

Older individuals frequently experience reversible “frailty syndrome,”, increasing incidence of disability. Although physical exercise interventions may delay functional decline, there are difficulties in implementing them and performing seamless follow-up at home. Very few technological solutions attempt to address this challenge and improve individual participation.

**Objective:**

Our objectives are to (1) develop a technological solution designed to support active aging of frail older persons, (2) conduct a first laboratory evaluation of the device, and (3) design a multidimensional clinical trial to validate our solution.

**Methods:**

We conducted a first phase of multidisciplinary meetings to identify real end users and health professional’s unmet needs, and to produce specifications for the architecture of the solution. In a second phase, we performed laboratory tests of the first proposed prototype (a smart insole) with 3 healthy volunteers. We then designed an ongoing clinical trial to finalize the multidimensional evaluation and improvement of the solution.

**Results:**

To respond to the needs expressed by the stakeholders (frailty monitoring and adherence improvement), we developed a prototype of smart shoe insole to monitor key parameters of frailty during daily life and promote walking. It is a noninvasive wireless insole, which automatically measures gait parameters and transmits information to a remote terminal via a secure Internet connection. To ensure the solution’s autonomy and transparency, we developed an original energy harvesting system, which transforms mechanical energy produced by the user’s walking movement into electrical energy. The first laboratory tests of this technological solution showed good reliability measures and also a good acceptability for the users. We have planned an original iterative medical research protocol to validate our solution in real life.

**Conclusions:**

Our smart insole could support preventive strategies against disability in primary care by empowering the older patients without increasing the busy health professional’s workload.

**Trial Registration:**

Clinicaltrials.gov NCT02316600; https://clinicaltrials.gov/ct2/results?term=NCT02316600&Search=Search. Accessed: 2015-05-13 . (Archived by WebCite at http://www.webcitation.org/6YUTkObrQ).

## Introduction

Many older individuals experience progressive functional decline despite the absence of a clear causal disease. This process has been labeled clinically as the “frailty syndrome” [1], which is characterized by a decrease in the capacities needed by an individual to adequately face stressors. To translate the theoretical concept of frailty into practice, Fried et al [1] proposed a model combining the evaluation of five criteria, namely, muscle weakness, self-reported exhaustion, unintentional weight loss, low physical activity, and slow gait speed. Although frailty is a multimodal syndrome, gait speed is recognized as a global indicator of health in older persons [2]. Several authors have evaluated gait speed as a predictor of future disability, mortality, institutionalization [3-5], and health-related events, even those apparently disconnected to physical function, such as cognitive impairment [6,7].

Frailty is potentially reversible [1], and a number of healthy lifestyle interventions can now be proposed [8-10]. Nevertheless, there are difficulties in implementing long-term preventive interventions, obtaining satisfactory patient adherence, and carrying out seamless follow-up of frail older persons at home.

The use of technology could be relevant for frailty assessment [11], as well as for promoting and monitoring exercise at home [12-14] and predicting health-related events [15]. Computer-based exercise interventions administered via a telecommunications system at home seem to be efficient [16,17], and remote feedback in home-based physical activity interventions seems to be as effective as supervised exercise interventions [18]. Monitoring could potentially, in itself, improve adherence and performances [13]. “Quantified self” devices are already proposed to young robust people to encourage their adherence and motivation (eg, Fitbit, Nike+). By contrast, in current clinical practice, no devices are used to measure activity and gait speed and give feedback to the patient. In fact, follow-up of frail elderly patients is almost nonexistent. However, to reach our goal we need to make more accurate measurements, especially concerning gait speed, and to make it less obtrusive for the end users. Given the importance of the relationship between gait speed at usual pace and risk of adverse events, and because of the amount of change for a 0.1-m/s variation [19], we are seeking 0.1-m/s accuracy, which is not provided by commercial devices and mobile phones. In addition, to ensure long-term acceptability it is important to develop a discreet, transparent, and self-reliable device. Practically, this means that there should be no need for direct human intervention in data transmission and battery charging. That is far from being the case with commercial devices and mobile phones delivered today.

We believe that there is a specific need for the development of a patient-centered device that provides accurate and unobtrusive assessment of physical activity and gait speed, as well as intervention adherence through feedback and self-motivation. Our overall objective is to develop and validate a smart device to support healthy aging of frail older persons (ClinicalTrials.gov identifier NCT02316600).

## Methods

We conducted a first phase of medical and technical specifications to develop a device that will address unmet needs of frailty. This first phase involved medical practitioners (gerontologists) from the Toulouse University Hospital (France), who identified unmet medical needs in clinical frailty management. A systematic literature review of technologies for frailty and disability was performed [20], and a focus group was conducted to come to a consensus. The main unmet needs reported from the clinical ward were as follows: the difficulty to follow-up on patients’ frailty indicators in the community setting and to obtain adherence through feedback and motivational coaching. This phase also involved researchers from the Laboratory for Analysis and Architecture of Systems-Centre National de la Recherche Scientifique (LAAS-CNRS) who explored technical opportunities and locks. We then built a consortium to address the device development challenge.

In a second phase, we developed the first prototype of the device (a smart insole) with several partners, including new technologies companies (metrological tools and remote monitoring), podiatrist experts, LAAS-CNRS, and clinical experts on frailty. We were rapidly able to perform laboratory tests of the first prototype with 3 healthy volunteers. This took place in the LAAS-CNRS living laboratory and was part of a multiphased multidimensional trial. The first complete technical laboratory phase, which is not described here, did not include clinical tests and was centered on technical performances of the prototype. The acceptability, security, and performances of the solution are evaluated at each phase of the iterative clinical evaluation, which is fully accepted by the regional medical ethic committee.

## Results

### Smart Insole Specifications

According to the unmet needs reported from the clinical ward, we were able to resume the medical specifications for the device as follows:

- The device should target the community setting and more specifically the follow-up of the patients;
- The device should measure major physical frailty criteria, such as muscle weakness, unintentional weight loss, low physical activity, and slow gait speed;
- The device should not only perform a seamless follow-up but should also support adherence to recommendations;
- The device should be unobtrusive and need no maintenance;
- The overall solution has to be patient centered, avoiding work overload for the medical practitioner;
- The device should provide patient feedback regarding performances, evolution of health status, and alerts in case of abnormal trends in indicators through existing terminals, such as touchpads or via mobile phones;
- The device should provide a feedback to the medical practitioner through the Internet, including history of health status indicators alerts according to prespecified thresholds and adherence to recommendations.

These medical specifications may express wishes that could not be fulfilled from a technical point of view. This phase also involved researchers from the LAAS-CNRS who explored the technical possibilities and locks. With regard to medical specifications, technical possibilities, and on the basis of previous works [21], we chose to develop a wireless smart shoe insole, which would meet technological specifications, while providing good acceptability and unobtrusiveness for the end users. This device should be able to measure the following:

- Activity periods and their durations over time;
- For each activity period, the number of steps, average speed, and distance covered were calculated;
- Energy expenditure (indirect data);

Unintentional weight loss and muscle weakness will not be directly measured with this first insole prototype. We will have to use other means such as direct questioning via the Web-based interface.

We conducted two sessions with end-user focus group (12 robust old adults) to validate this choice. Given the technical specifications, the consortium—new technologies firms (metrological tools and remote monitoring), podiatrist experts, LAAS-CNRS, and clinical experts on frailty—was able to address the device development challenge.

### Prototype Design

**Figure 1:**
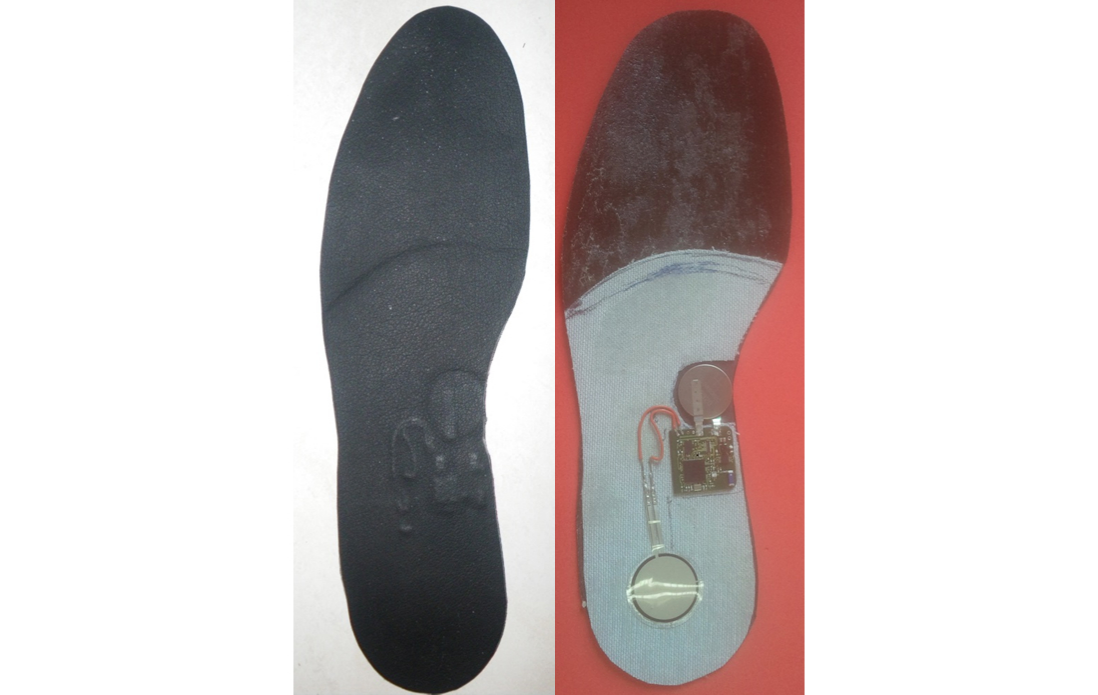
Smart insole.

**Figure 2:**
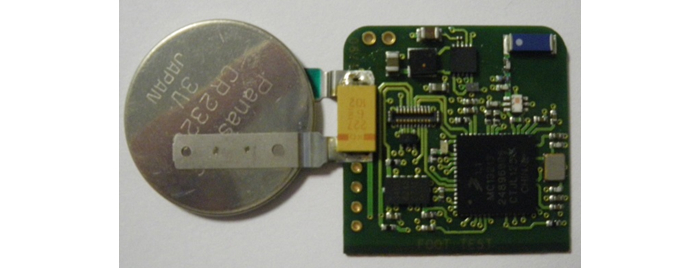
Sensors tag.

**Figure 3:**
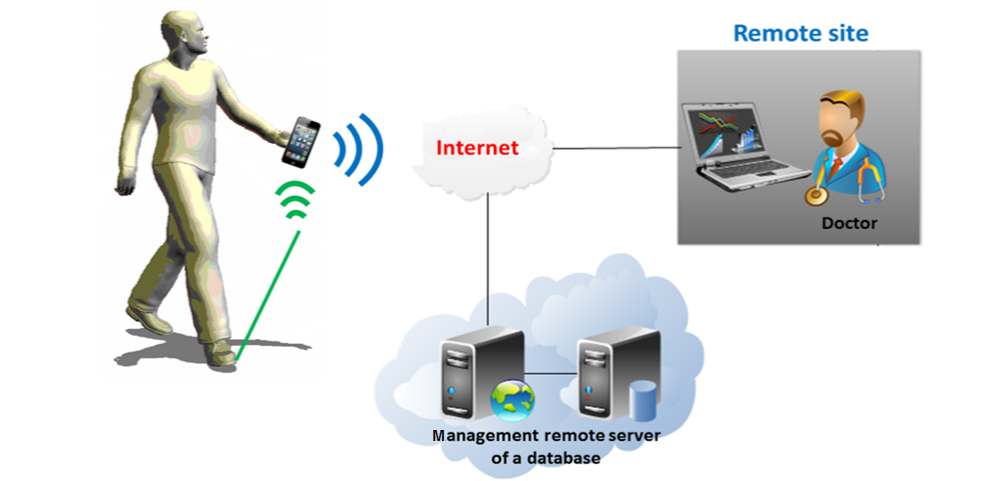
Operational setup.

In the second phase, we developed the first prototype of the smart insole (Figure 1) including our sensors tag (Figure 2). It was designed by the consortium at the LAAS-CNRS laboratory. Developing an efficient means of powering the device was an important consideration during the design process, thus enabling full autonomy and transparency. Accelerometers and gyroscopes are the most frequently used types of sensors to measure the gait characteristics [22,23]. In our device, we used an accelerometer because it is more compact and consumes less power than a gyroscope. The printed circuit board embedded in the insole includes the following elements: a low-power 3-axis acceleration sensor, a global system comprising a low-power microprocessor unit and a transceiver, a flash memory for local data logging, and a nano-powered time keeper to activate scheduled data-logging modes. This system measures gait parameters when the accelerometer detects an activity. The dimensions of the system are 3.2 cm × 2.2 cm × 2.0 mm and total weight is 5 g, including the battery. The smart insole will be part of an operational setup illustrated in Figure 3. The following components are included in our system:

- A radio beacon for automatic data collection (when the wireless insole detects the beacon);
- A collection terminal with an Internet connection (touchpad, mobile phone);
- A remote server for database management; and
- A Web application used by the person and his/her physician via a remote access.

In its current version, a lithium battery CR2016 supplies the smart insole with a capacity of 90 mAh. An energy harvesting system is also proposed aiming to produce an unobtrusive self-powered insole. Piezoelectric generators transform mechanical energy produced by the user’s walking movement into electrical energy.

**Figure 4:**
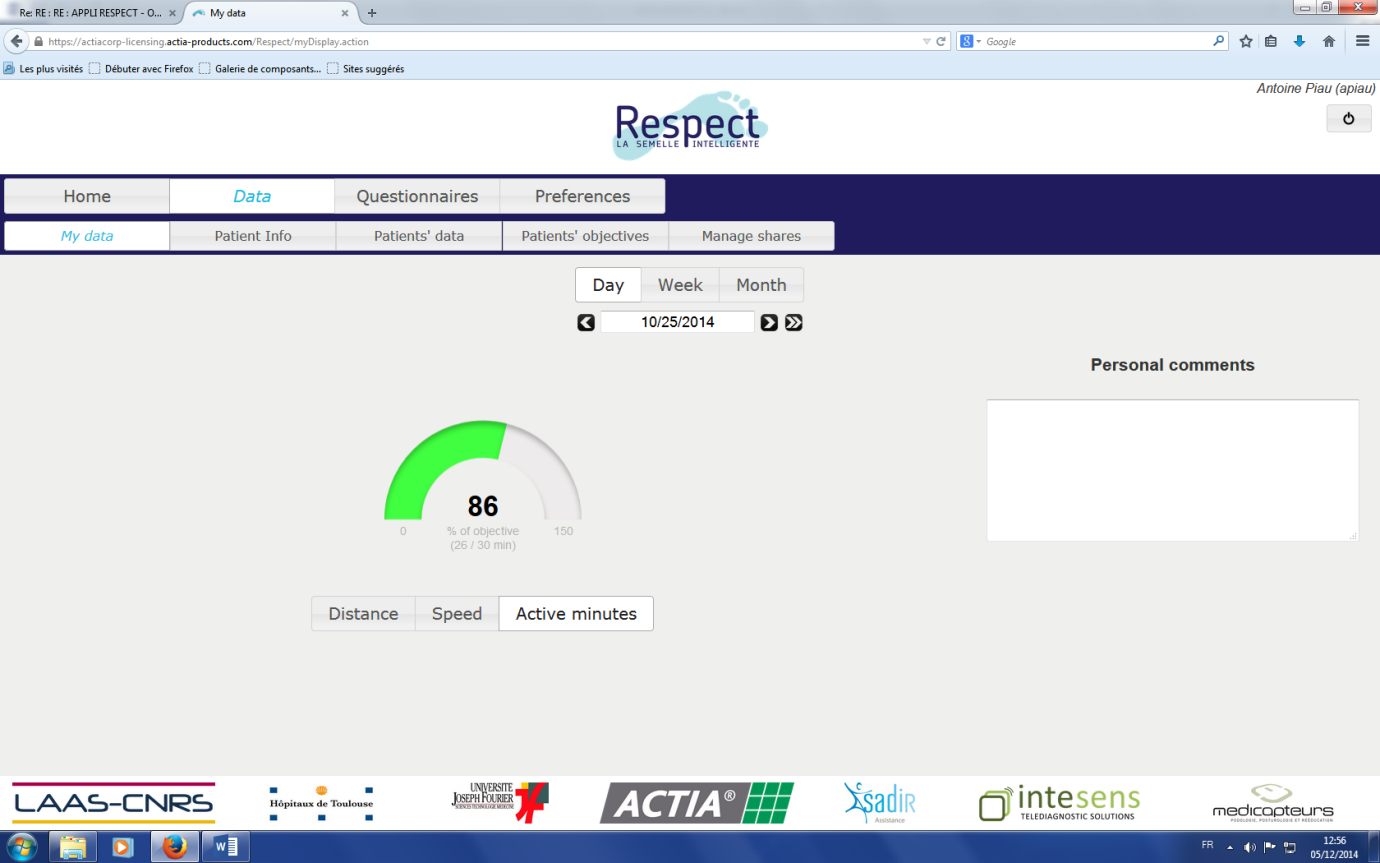
End user interface: active minutes.

**Figure 5:**
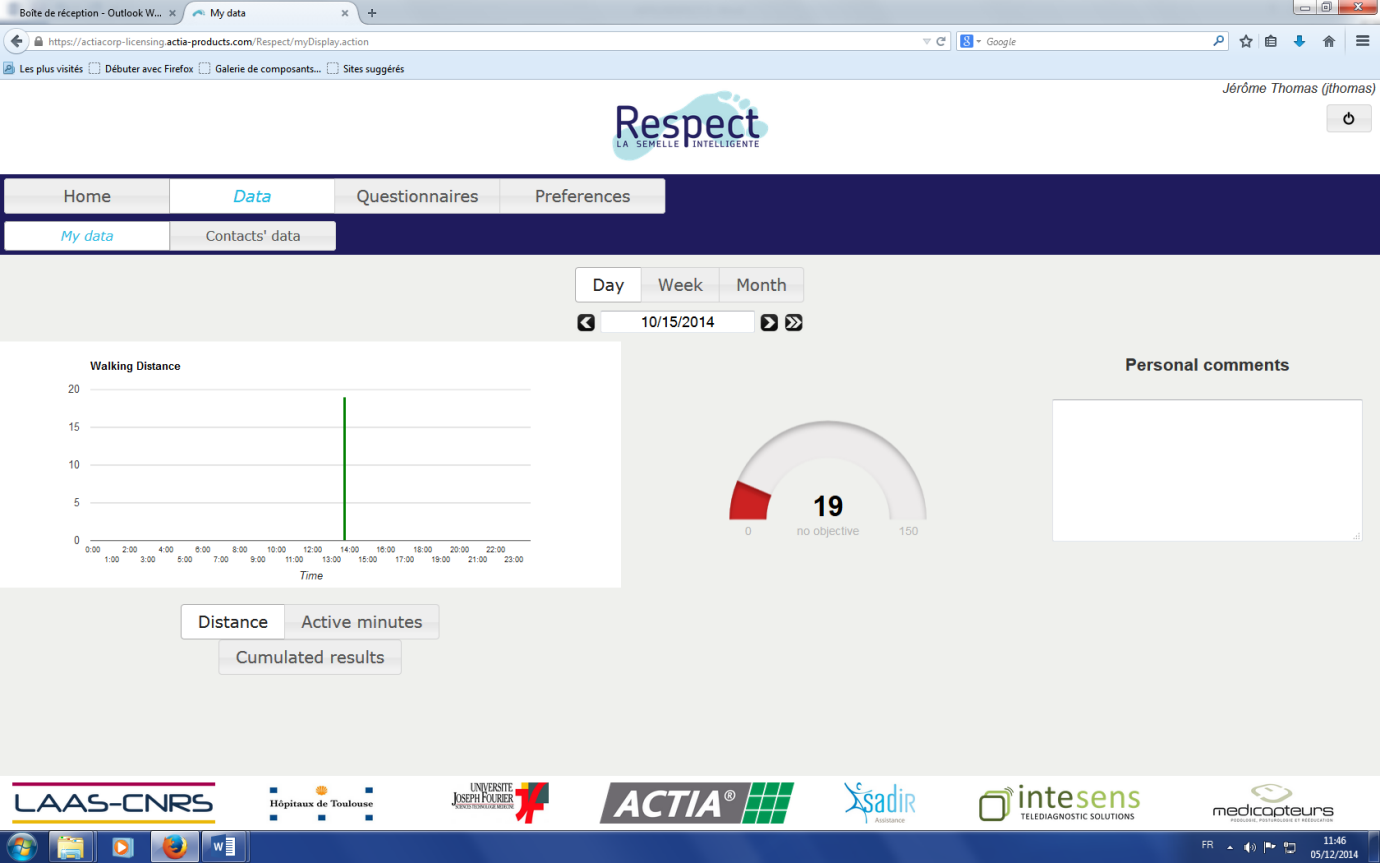
End user interface: distance.

**Figure 6:**
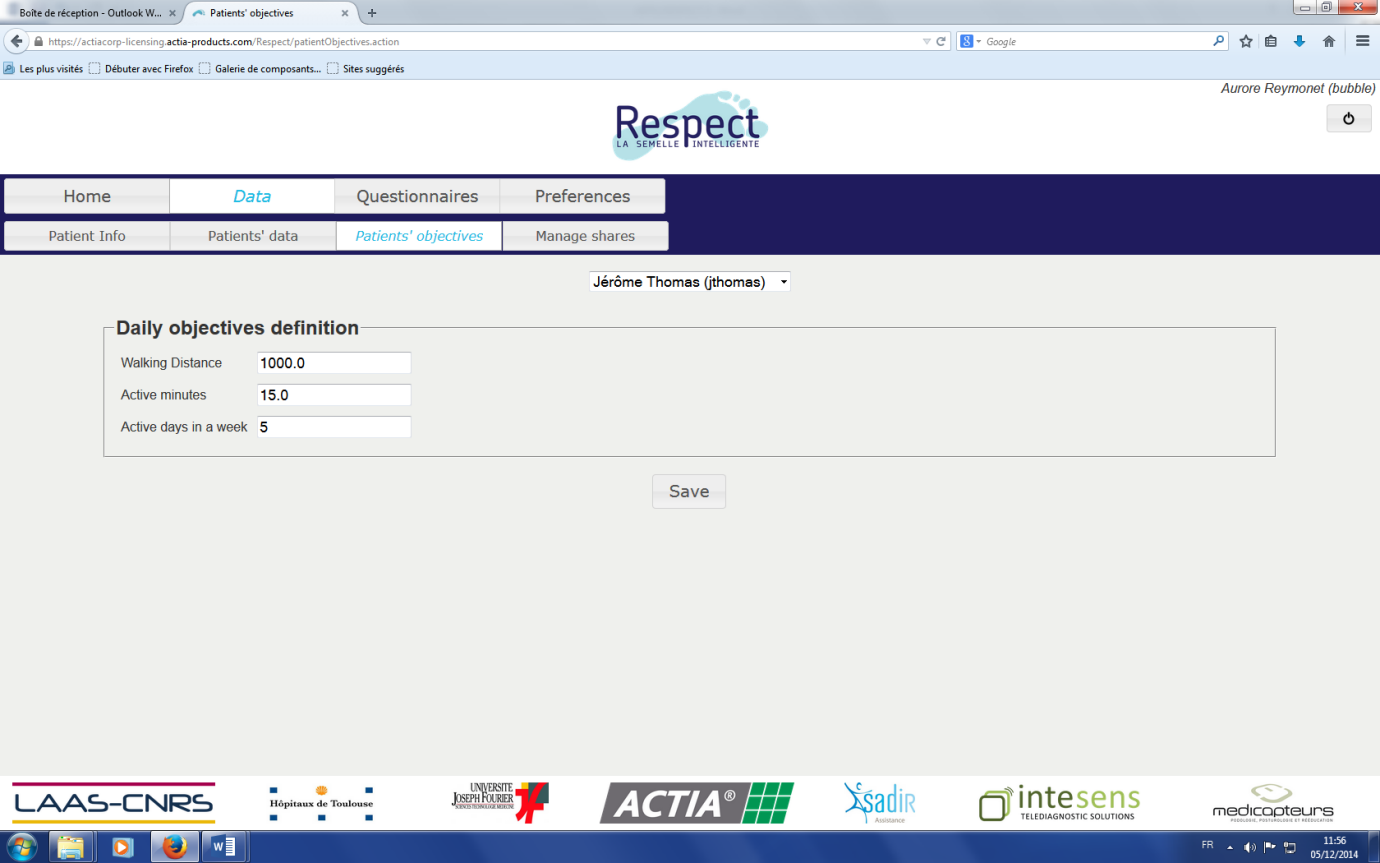
Physician interface.

This solution monitors several frailty indicators and feedback is given to the user during his/her daily life at home. A screen capture of the end-user Web application is shown in Figures 4 and 5. This will enable the user to be informed about self-adherence with respect to individual physical exercise objectives, personal health status evolution, and a possible alert in case an abnormal trend in indicators occurs. The physician interface is presented in Figure 6. This first prototype does not include weight sensors, which is currently under development but not yet consolidated.

### Preliminary Living Laboratory Findings

#### Overview

We performed preliminary laboratory tests in 3 healthy volunteers in the LAAS-CNRS living laboratory, to validate the stride detection algorithm for gait speed monitoring before larger clinical trials, as well as to test the energy harvesting system. The volunteers conducted tests on a treadmill by following three-step instructions (slow, medium, and fast).

#### Gait Speed Monitoring

**Figure 7:**
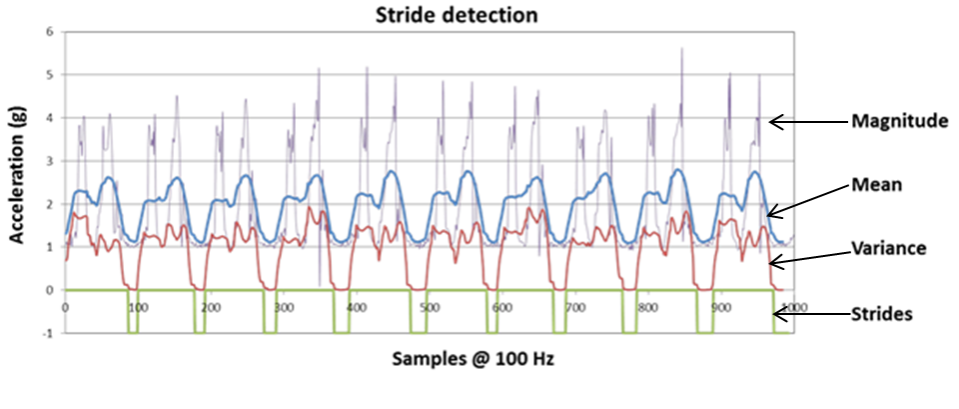
Stride detection process.

Accelerometers and gyroscopes are mainly used in the literature [22,24,25] to measure the dynamic characteristics of walking from the foot position. In our device, we used an accelerometer because it is more compact and has more low power consumption than a gyroscope. For stride detection with low-cost accelerometer, Jimenez et al [26] proposed a reliable algorithm with an error of 0.1% for a normal gait speed. We added a method to measure cadence. The accelerometer was set to capture sensor samples at 100 Hz. The algorithm implemented for stride detection and cadence measurement includes the following five steps:

- Compute the magnitude of the acceleration;
- Compute the local mean acceleration value;
- Compute the local acceleration variance, to highlight foot activity and to remove gravity;
- Stride detection with two thresholds on the local acceleration variance: the first threshold detects the rising edge, and the second threshold detects the falling edge (Figure 7); and
- After stride detection, we use it to compute the local cadence expressed in steps/second (1 stride is equivalent to 2 steps) with a sliding windows on the last 3 strides (6 steps).

This stride detection algorithm was implemented in the smart insole. Measurements were performed on a treadmill to determine the robustness of this method. A total of 3 volunteers (men aged 25, 29, and 30 years) were requested to walk at 5 gait speeds on the treadmill, which were fixed (0.5, 0.75, 1.0, 1.25, and 1.5 m/s). For each gait speed, 100 strides were performed. The results for each volunteer are presented in Table 1.

Errors were observed on less than 1% of the number of strides. Less than 1% of error on the number of strides was observed over the entire speed range studied (0.5-1.5 m/s for each step of 0.25 m/s). It was also reported that the measured cadence was relatively stable for a constant gait speed, as cadence variations were approximately 1% for a stable gait speed, over the gait speed range studied. These preliminary tests showed a strong correlation between gait speed and cadence. It seems possible to assess walking speed in an ambulatory setting by measuring cadence, when an individual’s specific relationship between cadence and gait speed is established. For this purpose, training would need to be implemented to calculate this relationship. A specific tool was designed to automatically calibrate the measurements of the insole during the training period. This system is based on the use of two light barriers (infrared transceiver combined with an optical reflector and a transmitter) that measure true mean gait speed over a distance of 4 m.

**Table 1:** Test of the stride detection algorithm.

Gait speed (m/s)		Real number of strides	Number of strides counted by the smart insole	Percentage of errors
**Volunteer 1**				
	0.5	100	102	2
	0.75	100	101	1
	1	100	100	0
	1.25	100	100	0
	1.5	100	99	1
	Total	500	502	0.4
**Volunteer 2**				
	0.5	100	99	1
	0.75	100	99	1
	1	100	100	0
	1.25	100	99	1
	1.5	100	99	1
	Total	500	496	0.8
**Volunteer 3**				
	0.5	100	98	2
	0.75	100	100	1
	1	100	100	0
	1.25	100	100	1
	1.5	100	99	1
	Total	500	497	0.6

#### Energy Harvesting System

Previous studies have shown that for a 68-kg person walking at a speed of 2 steps/second with a heel movement of 5 cm, the maximum power that can be generated is 67 W [27]. Only thin-film piezoelectric generators can be integrated in the thickness of an insole, such as lead zirconate titanate and polyvinylidene fluoride piezoelectric materials [28]. We tested the feasibility of an energy-autonomous smart insole based on the solution proposed by Smart Material Corporation. For a rapid walking speed (1.5 and 1.75 m/s), energy needs are covered. For a slow walking speed (0.5 m/s), one third of the energy needs are covered. The feasibility stage is conclusive and enables us to launch the design stage of a miniaturized energy harvesting system.

## Discussion

### Principal Findings

The population is aging rapidly. Yet, added life years are not always lived in healthy conditions and independency. A major goal of aging interventions is not only to extend life, but also to preserve the capacity for independent living. Frailty is considered as a predisability state which, unlike disability, is still amenable for interventions. This new concept of frailty modifies the common geriatric approach by leading it toward the importance of prevention. Nevertheless, we still have difficulties to implement long-term preventive interventions against disability, to obtain the adequate patient’s adherence and participation, and to perform a seamless and efficient follow-up of older persons at home.

Although information and communication technologies (ICTs) have proven their efficiency for monitoring various chronic diseases such as heart failure or diabetes, very limited evidence is available about the application of technologies in early prevention of physical disability [29,30]. According to a review by Marziali et al [31], only 1% of the home health care programs studied focused on this situation. Nevertheless, using technology in this direction does make sense. A review of 2246 publications [32] has demonstrated that quantified self-tools can motivate sedentary individuals to change their habits. ICT, as visiophonic communication, could also be helpful for intervention implementation [33]. Technologies may support intervention at home and prevent negative health-related outcomes by detecting early signs of deteriorating health.

We designed a technological tool to support continuous monitoring of key parameters of frailty. The first version of our unobtrusive insole allowed us to implement the algorithms of the dynamic gait parameters assessment.

In our study, the stride detection method was robust and accurate over the entire speed range studied with healthy young volunteers. A strong correlation was reported between walking speed and cadence and the feasibility of the energy harvesting system. These tests must be carried out in natural walking conditions with senior end users, which will present walking patterns potentially impeding these results. Even if each individual’s specific relationship between cadence and gait speed is established, the relationship could change over time. The uncertainty about the evolution of the relationship between gait speed and cadence in a prefrail and frail elderly population is one of the most important limitations of our project. Scientific data are scarce, but according to a recent review [34], the prominent parameter related to prefrailty is reduced cadence, whereas frailty (vs prefrail status) is characterized by reduced step length in everyday walking. This could potentially drive the necessity to repeat personal calibration process over time. These initial results show the feasibility of an instrumented insole, which is an energy autonomous device (energy harvest and energy generation). Indeed, for a rapid walking speed (1.5 and 1.75 m/s), the energetic needs are covered. In case of slow walking speed (0.5 m/s), one third of the energetic needs are covered. A simple solution to design a more efficient energy harvesting system is to use a piezoelectric generator with a larger active area or to use multiple stacked piezoelectric generators. To cover all the ranges of walking speed, we are also reducing the power consumption of the insole.

Lastly, based on the Toulouse University Hospital method for medical device evaluation, we designed a two-phase original clinical trial to finalize the multidimensional evaluation of the smart insole. Although there is no consensus for health technologies evaluation, several methods have been proposed [35,36]. They all emphasize the multidimensional aspect and the iterative character of the evaluation. The first phase of our testing was centered on gait speed measurement and energy harvesting. We are currently performing two additional phases of multidimensional evaluation thanks to a national grant from the French National Research Agency (Project Number ANR-13-TECS-0007). The first clinical and technical feasibility trial will include 15 healthy old people to evaluate the acceptability and the technical performances of the first prototype (from October 2014 to December 2015). In parallel with this first clinical phase, we are planning to make technical improvements to the insole by adding an additional frailty parameter measurement: weight monitoring, and by developing a touchpad motivational coaching software through the Internet. Then, the final solution (shoe insole and coaching software) will be evaluated in a larger clinical comparative study to assess its acceptability during field tests in real-life conditions. This phase will include 60 frail individuals living at home (from January 2015 to June 2016). We included a new partner for the acceptability evaluation: Age-Imaging-Modelization Laboratory of the Joseph Fourier University (Grenoble, France), a new research laboratory devoted to the science and technology of aging. We believe that our solution has to be patient centered. Our aim is not to propose telehealth, which would lead to additional costs because of the need for a dedicated helpline. Nevertheless, the solution provides the physician with additional useful information (health status and adherence to recommendations) without interfering with the organization of health care. Access to seamless follow-up could lead to earlier diagnosis and prevention of the high burden of disability. This project also addresses global issues relating to our centralized health system. There is a potential benefit for the frail older persons in adopting this kind of ICT solution, because of their active involvement in a healthy lifestyle project.

### Conclusion

Our purpose is to design a technological tool to support continuous monitoring with minimal invasiveness both at home and in the outside environment. This could be potentially helpful to promote healthy lifestyle recommendations in the frail older population. A first prototype has been developed in the living laboratory and has passed through a test phase involving volunteers. We are planning additional phases of multidimensional evaluation in real-life conditions.

## Abbreviations

ICT: information and communication technology
LAAS-CNRS: Laboratory for Analysis and Architecture of Systems-Centre National de la Recherche Scientifique
